# The German Version of the Mobile App Rating Scale (MARS-G): Development and Validation Study

**DOI:** 10.2196/14479

**Published:** 2020-03-27

**Authors:** Eva-Maria Messner, Yannik Terhorst, Antonia Barke, Harald Baumeister, Stoyan Stoyanov, Leanne Hides, David Kavanagh, Rüdiger Pryss, Lasse Sander, Thomas Probst

**Affiliations:** 1 Clinical Psychology and Psychotherapy Ulm University Ulm Germany; 2 Clinical and Biological Psychology Catholic University Eichstaett-Ingolstadt Eichstaett-Ingolstadt Germany; 3 Creative Industries Faculty Queensland University of Technology Brisbane Australia; 4 Faculty of Health and Behavioural Sciences University of Queensland Brisbane Australia; 5 Psychology and Counselling Queensland University of Technology Brisbane Australia; 6 Institute of Databases and Information Systems Ulm University Ulm Germany; 7 Department of Rehabilitation Psychology and Psychotherapy University of Freiburg Freiburg Germany; 8 Department for Psychotherapy and Biopsychosocial Health Danube University Krems Krems Austria

**Keywords:** mHealth, Mobile App Rating Scale, mobile app, assessment, rating, scale development

## Abstract

**Background:**

The number of mobile health apps (MHAs), which are developed to promote healthy behaviors, prevent disease onset, manage and cure diseases, or assist with rehabilitation measures, has exploded. App store star ratings and descriptions usually provide insufficient or even false information about app quality, although they are popular among end users. A rigorous systematic approach to establish and evaluate the quality of MHAs is urgently needed. The Mobile App Rating Scale (MARS) is an assessment tool that facilitates the objective and systematic evaluation of the quality of MHAs. However, a German MARS is currently not available.

**Objective:**

The aim of this study was to translate and validate a German version of the MARS (MARS-G).

**Methods:**

The original 19-item MARS was forward and backward translated twice, and the MARS-G was created. App description items were extended, and 104 MHAs were rated twice by eight independent bilingual researchers, using the MARS-G and MARS. The internal consistency, validity, and reliability of both scales were assessed. Mokken scale analysis was used to investigate the scalability of the overall scores.

**Results:**

The retranslated scale showed excellent alignment with the original MARS. Additionally, the properties of the MARS-G were comparable to those of the original MARS. The internal consistency was good for all subscales (ie, omega ranged from 0.72 to 0.91). The correlation coefficients (r) between the dimensions of the MARS-G and MARS ranged from 0.93 to 0.98. The scalability of the MARS (H=0.50) and MARS-G (H=0.48) were good.

**Conclusions:**

The MARS-G is a reliable and valid tool for experts and stakeholders to assess the quality of health apps in German-speaking populations. The overall score is a reliable quality indicator. However, further studies are needed to assess the factorial structure of the MARS and MARS-G.

## Introduction

Mobile phones are an integral part of modern life. In Europe, 67% of the population owns smartphones, and the number of smartphone users is rising worldwide [1]. It has been reported that 30% of Germans have 11 to 20 apps installed on their smartphones [2]. The use of mobile apps to improve mental health and well-being is becoming increasingly common, with roughly 29% of Germans currently using at least one health app [3].

Globally, between 95 million and 130 million people speak German, making it the 11th most spoken language worldwide [4,5]. Elderly individuals and people with basic education are commonly monolingual in Germany [6]. Yet, these populations have a high need for assistance in developing and maintaining health behaviors and could benefit from the use of mobile health apps (MHAs). A German MHA rating scale could help researchers and health care providers assess the quality of health apps quickly and reliably in their mother tongue. Furthermore, it would be easy to rate a German app with a German scale.

MHAs offer unique and diverse possibilities for health promotion. They allow ecological momentary assessments [7,8] and interventions [8,9]. Additionally, they can be used irrespectively of geographical, financial, and social conditions; can simultaneously target nonclinical and clinical populations; and have the capacity to provide diverse health-management strategies in an ecological setting [10]. Moreover, they support individuals, including those from high-need high-cost populations (eg, those with chronic or lifestyle diseases), in managing their health [9]; reduce help-seeking barriers; and offer a wide range of engagement options [10].

Despite the recent proliferation of MHAs [11], there are no universally accepted criteria for measuring and reporting their quality [8,12]. Therefore, it is necessary to support researchers, users, and health care providers (eg, physicians, psychotherapists, and physiotherapists) in selecting high-quality MHAs. Safe and reliable use of MHAs requires evidence of efficacy and quality, information about data protection, information about routines for emergencies (eg, self-harm and adverse effects), and overall consideration of associated risks [10].

Boudreaux and colleagues [13] suggested the following seven strategies to evaluate MHA quality: (1) Review the scientific literature; (2) Search app clearinghouse websites; (3) Search app stores; (4) Review app descriptions, user ratings, and user reviews; (5) Conduct a social media query within professional and, if available, patient networks; (6) Pilot the apps; and (7) Elicit feedback from users. This process might be too demanding for health care providers and end users when making treatment choices. A standardized and reliable quality assessment tool could facilitate this process.

Several MHA evaluation scales exist to date. The American Psychological Association released an app evaluation model comprising 33 items across the following five scales: background information, risk/privacy and security, evidence, ease of use, and interoperability [12]. The main aim of this model is to assess the likelihood of harm [9]. However, the validity and reliability assessment of this rating instrument has not yet been reported, and there is no agreement regarding its application [14].

Baumel and colleagues [15] developed the Evaluation Tool for Mobile and Web-Based eHealth Interventions (ENLIGHT), according to a comprehensive systematic review of relevant criteria. The tool allows the evaluation of app quality in terms of seven dimensions (usability, visual design, user engagement, content, therapeutic persuasiveness, therapeutic alliance, and general subjective evaluation) with 28 items. ENLIGHT also provides a checklist to assess credibility, evidence base, privacy explanation, and basic security.

The Mobile App Rating Scale (MARS) [16] is the most commonly used app evaluation tool that allows electronic health experts to rate MHAs. It includes 19 items comprising four subscales on objective MHA characteristics (engagement, functionality, esthetics, and information quality) and a further 10 items comprising two subscales on subjective characteristics (subjective app quality and perceived impact). The subscale and overall scores indicate the quality of MHAs. The MARS has been used to scientifically assess app quality in the following fields: weight management, physical activity, heart failure, diet in children and adolescents, medication adherence, mindfulness, back pain, chronic pain, smoking cessation, and depression [8,17-24]. Thus, it is the most widely used MHA quality rating tool in the scientific community. Furthermore, numerous international efforts promoting safe MHA use (eg, Mobile Health App Database, PsyberGuide or App Script, Reachout, Kinds Helpline, Health Navigator, and Vic Health) are based on the MARS.

The original version of the MARS is in English, but culture- and language-specific app ratings are needed globally. Spanish and Italian versions of the MARS have been developed [25,26]. A German MARS is necessary considering the growing and unregulated MHA market in Germany. Therefore, this study aimed to develop and validate a German version of the Mobile App Rating Scale (MARS-G) and to investigate the scalability of the overall MARS score with Mokken scale analysis—an approach that is closely related to item response theory.

## Methods

### Adaptation and Translation

The MARS was translated from English into German by two independent bilingual scientists (EMM and TP). After review and discussion of both forward translations, a pilot version of the MARS-G was created. This pilot version underwent blind back translation by two bilingual speakers with different backgrounds (a postdoctoral psychologist [AB] and a nonacademic individual [LMZ]). Thereafter, the back translation was compared with the original English version by the bilingual scientists (EMM and TP), and the penultimate version of the MARS-G was created. This version was evaluated for comprehensibility by three researchers and three nonacademics. After addressing their comments, the final version of the MARS-G was created and used in this study. The MARS-G can be downloaded from the supplementary materials or obtained from the authors on request.

### Search and Procedure

The MARS-G was validated within the framework of a study on the quality of apps targeting anxiety (E M Messner et al, unpublished data, 2020). Apps were identified using the following search terms: anxiety, fears, anxiety attack, anxious, anxiousness, anxiety disorder, fear, dread, fearful, panic, panic attacks, worry, and worries. Each search term was provided separately, as no truncation or use of logic operators (AND, OR, and NOT) was possible in the Google Play Store and iOS Store.

The inclusion process was divided into three steps (searching, screening, and determining eligibility). (1) Using the search terms mentioned above, the initial app pool was identified. (2) App details on the store sites were screened, and apps were downloaded and reviewed if they were developed for anxiety, were available in German or English, were downloadable through the official Google Play Store or iOS Store, and met no relevant exclusion criteria (app bundles [many applications only available as a group]). (3) All downloaded apps were assessed and excluded if they did not address anxiety, were not in German or English, were malfunctioning, or met relevant exclusion criteria (device incompatibility and development/test phase). We identified 3562 MHAs from the app stores. However, we excluded 810 duplicate apps, 2577 apps considered inappropriate on screening, and 71 apps considered ineligible. The remaining 104 apps were rated using the MARS and MARS-G by two independent trained raters. The raters tested all MHAs for 15 minutes. Quality was assessed immediately after the testing period in both languages. The assessment of the MARS-G is present in a review evaluating the quality of MHAs available for anxiety (E M Messner et al, unpublished data, 2020).

### Rater Training

We followed the rating methodology in the original study by Stoyanov and colleagues [16]. We created a YouTube video with an introduction on MARS-G rating and an exercise on how to rate an app used as an exemplary health app (TrackYourTinnitus) [27]. This video can be requested from the corresponding author. Each rater was trained using this video, and five predefined apps were then rated to ensure that each rater was appropriately trained. If the individual rating score was different from our standard rating score by at least 2 points, the difference was discussed until agreement. All raters had at least a bachelor’s degree in psychology to ensure a necessary minimum psychodiagnostic competence standard.

### German Version of the Mobile Application Rating Scale

We added the following items in the app description section for the MARS-G: theoretical background (cognitive-behavioral, therapy, systemic therapy, etc), methods (eye movement desensitization and reprocessing, tracking, feedback, etc), category in the app store (lifestyle, medicine, etc), embedding into routine care (communication with therapist, etc), type of use (prevention, treatment, rehabilitation, etc), guidance (stand-alone, blended care, etc), certification (medical device law, etc), and data safety (log in, informed consent, etc). The four sections of the original MARS were expanded with an additional section focusing on the therapeutic gain associated with the app. The derived items were as follows: gain for the patient; gain for the therapist; risks and adverse effects; and ease of implementation in routine health care.

### Analyses

#### Intraclass Correlation

The included MHAs were rated independently by two trained raters. The intraclass correlation coefficient (ICC) was calculated to assess the extent of agreement between the raters. An ICC of <0.50 indicated poor correlation, 0.51-0.75 indicated moderate correlation, 0.76-0.89 indicated good correlation, and >0.90 indicated excellent correlation [28]. According to the findings of previous studies, an ICC >0.75 was considered to indicate sufficient correlation [8,29,30].

#### Internal Consistency

Internal consistency of the MARS-G and its subscales was assessed as a measure of scale reliability, similar to the original MARS [16]. Omega was used instead of the widely adopted Cronbach alpha to assess reliability, as it provides a more unbiased estimation of reliability [31-33]. For estimations, the procedure introduced by Zhang and Yuan [34] was used to obtain robust coefficients and bootstrapped bias-corrected confidence intervals. Reliability of ω <0.50 indicated unacceptable internal consistency, 0.51-0.59 indicated poor consistency, 0.60-0.69 indicated questionable consistency, 0.70-0.79 indicated acceptable consistency, 0.80-0.89 indicated good consistency, and >0.90 indicated excellent consistency [35].

#### Validity

We assessed correlations between corresponding subscales of the MARS and MARS-G, as well as the overall correlation between the MARS total score and MARS-G total score. A *r* value >0.8 was a priori considered by the author group as an indicator of a strong and sufficient association between the MARS and MARS-G. Additionally, mean comparisons were performed between the dimensions of the MARS and MARS-G, using two-sided *t* tests. For all comparisons, a *P* value <.05 was considered significant.

#### Mokken Scale Analysis

Mokken scale analysis (MSA) is a scaling approach closely related to nonparametric item response theory [36]. The preconditions to use MSA are monotonicity and nonintersection. The key parameter in the MSA is Loevinger H. H_i_ is the scaling parameter for item i, and the overall scalability of all items clustering onto scale k is H_k_. H_i_ indicates the strength of the relationship between a latent variable (app quality) and item i. A high scalability score indicates a high probability that an increase in item i is accompanied by an increase in the latent variable. A scale is considered weak if H is <0.4, moderate if H is ≥0.4 but <0.49, and strong if H is >0.5 [36]. This approach has been described in detail previously [36-39]. For both the MARS and MARS-G, the MSA was conducted to assess the scalability of the mean scores. As recommended by van der Ark [36], the reliability of the scales was additionally assessed using the Molenaar-Sijtsma method (MS) [40,41], lambda-2 [42], and latent class reliability coefficient (LCRC) [43]. The MSA has been described previously [36].

#### Analysis Software

R software (R Foundation for Statistical Computing, Vienna, Austria) [44] was used for all analyses, except intraclass correlation. The MSA was conducted using the R package *mokken* [36,38]. Correlations and internal consistency were calculated using the *psych* (version 1.8.12) [45] and *coefficientalpha* packages (version 0.5) [34]. The *coefficientalpha* package includes the calculation of omega with missing and nonnormal data. The ICC was calculated using IBM SPSS 24 (IBM Corp, Armonk, New York) [46].

## Results

### Descriptive Data and Mean Comparisons

The ICCs for the MARS and MARS-G were high (ICC_MARS_: 0.84, 95% CI 0.82-0.85; ICC_MARS-G_: 0.83, 95% CI 0.82-0.85). The mean and standard deviation scores of the items in the MARS-G are presented in Table 1. The mean and standard deviation scores of the items in the MARS are reported elsewhere (E M Messner et al, unpublished data, 2020). The mean scores of the dimensions *engagement* (*t*_206_=0.12; *P*=.91), *functionality* (*t*_205_=0.39; *P*=.70), *esthetics* (*t*_206_=−0.012; *P*=.99), and *information quality* (*t*_204_=0.45; *P*=.66) and the overall rating (*t*_206_=0.27; *P*=.80) were equivalent between the MARS and MARS-G.

**Table 1:** Summary of item and scale scores for the German version of the Mobile App Rating Scale.

Dimension		Score, mean (SD)
**Engagement**		**2.52 (0.70)**
	Item 01	2.64 (0.93)
	Item 02	2.79 (0.90)
	Item 03	2.19 (1.00)
	Item 04	1.86 (0.79)
	Item 05	3.15 (0.72)
**Functionality**		**4.12 (0.69)**
	Item 06	4.13 (0.82)
	Item 07	4.24 (0.77)
	Item 08	4.09 (0.74)
	Item 09	4.03 (0.78)
**Esthetics**		**3.21 (0.94)**
	Item 10	3.40 (0.93)
	Item 11	3.20 (1.09)
	Item 12	3.04 (0.99)
**Information quality**		**2.75 (0.60)**
	Item 13	3.60 (0.76)
	Item 14	2.63 (0.68)
	Item 15	2.67 (0.76)
	Item 16	2.61 (0.88)
	Item 17	3.66 (0.68)
	Item 18	1.87 (0.89)
	Item 19	3.00 (N/A^a^)
Overall mean		3.11 (0.58)

^a^This item on information quality could be rated for only 1 app, for the rest it was rated *not applicable*.

### Internal Consistency

The internal consistency for the MARS dimension *engagement* was good (ω=0.84, 95% CI 0.77-0.88). The internal consistencies for *functionality* (ω=0.90, 95% CI 0.85-0.94) and *esthetics* (ω=0.91, 95% CI 0.92-0.96) were excellent. The internal consistency for *information quality* was acceptable (ω=0.74, 95% CI 0.14-0.99; α=.75, 95% CI 0.67-0.83). The internal consistency of the overall MARS score was good (ω=0.81, 95% CI 0.74-0.86).

The internal consistencies of the MARS-G dimensions were almost identical to those of the original MARS (engagement: ω=0.85, 95% CI 0.78-0.89; functionality: ω=0.91, 95% CI 0.87-0.94; esthetics: ω=0.93, 95% CI 0.90-0.95; information quality: ω=0.72, 95% CI 0.33-0.81). The internal consistency of the overall score was good (ω=0.82, 95% CI 0.76-0.86).

### Validity

The correlation coefficients between corresponding dimensions of the MARS and MARS-G ranged from 0.93 to 0.98, and *P* values were adjusted for multiple testing according to the Holmes method [47] (Table 2). Correlations between the respective items are presented in Multimedia Appendix 1. There were no associations between user ratings and quality ratings (Table 1).

**Table 2:** Validity of the German version of the Mobile App Rating Scale (r and *P* value).

Dimension	Engagement_GER_^a^	Functionality_GER_	Esthetics_GER_	Information quality_GER_	Star rating
Engagement_ENG_^b^	0.97 (<.001)	0.49 (<.001)	0.73 (<.001)	0.52 (.001)	−0.03 (.99)
Functionality_ENG_	0.45 (<.001)	0.98 (<.001)	0.43 (<.001)	0.36 (.002)	0.06 (.99)
Esthetics_ENG_	0.69 (<.001)	0.41 (<.001)	0.97 (<.001)	0.41 (.001)	0.12 (.99)
Information quality_ENG_	0.55 (<.001)	0.34 (.004)	0.47 (<.001)	0.93 (.001)	0.25 (.19)
Star rating	−0.03 (>.99)	0.07 (>.99)	0.12 (>.99)	0.26 (.19)	—^c^

^a^German version.

^b^English version.

^c^Not applicable.

### Mokken Scale Analysis

The MSA of the MARS revealed strong scalability (H=0.50; SE 0.062). There were no violations of monotonicity and nonintersection. The internal consistency of this scale was acceptable (MS=0.74; lambda 2=0.73; LCRC=0.72). The MSA of the MARS-G revealed good scalability (H=0.48; SE 0.060). The internal consistency of this scale was acceptable (MS=0.74; lambda 2=0.72; LCRC=0.74). The scalability results of the MARS and MARS-G are presented in Table 3.

**Table 3:** Summary of the Hk coefficient (overall scalability of all items in the scale) for the Mobile App Rating Scale (MARS) and the German version of the Mobile App Rating Scale (MARS-G).

Dimension	MARS	MARS-G
Engagement	0.59	0.57
Functionality	0.43	0.41
Esthetics	0.51	0.51
Information quality	0.45	0.41
Total scale	0.50	0.48

## Discussion

### Principal Findings

This study developed and evaluated the MARS-G for MHAs. The results showed that the MARS-G is a reliable and valid tool for experts to assess the quality of MHAs. The validity and reliability of the MARS-G were comparable to those of the original MARS. With regard to the reliability of the dimension *information quality*, the confidence interval of omega was overestimated owing to planned missingness. The planned missingness originated from the response option *not applicable*, which allows raters to skip an item if the app does not have any health information (eg, diary apps and brain games). There were no differences in reliability between the MARS-G and original MARS.

The MSA revealed that the use of the MARS-G total score is appropriate. Furthermore, there was good correspondence between the MARS-G and original MARS, indicating good validity. Our results are consistent with the findings of a study that introduced and tested an Italian version of the MARS [25].

The MARS-G has been presented in Multimedia Appendix 2 and can be obtained from the authors on request. It can be used freely for research and noncommercial MHA-evaluation projects. To reach satisfactory interrater reliability, completion of an online training exercise provided by the corresponding author is highly recommended. Furthermore, a training dataset of five apps can be obtained from the corresponding author on request. The MARS-G ratings should be revised until an appropriate level (ie, ICC >0.75) of interrater reliability is achieved.

To assist in MHA selection, standardized high-quality ratings of MHA are needed in German-speaking countries. Overall, a publicly available database presenting reliable, valid, and standardized expert ratings, like MARS-G ratings, could contribute to informed health care decisions on which app to use for a specific disease or purpose. The mobile health app database [48] is one example of such a tool that assists users and health care providers in selecting appropriate apps for different health-related purposes.

### Limitations

This study has several limitations. First, convergent validity was only evaluated by comparing the MARS and MARS-G. Comparisons with other app rating scales, such as ENLIGHT [15] and the American Psychological Association app evaluation model [12], are necessary in future studies. Second, the focus on anxiety apps limits generalization. Further studies are needed to confirm that these findings can be generalized to other mobile health domains. Such studies would require expert raters who are familiar with the specific domain. Finally, a confirmatory factor analysis of the MARS and MARS-G should be conducted in future studies with larger samples to ensure that the predefined subscales of the MARS and MARS-G can be confirmed.

### Future Research

This translation study of the MARS led to the discovery of several research gaps. Future studies should focus on the improvement of app quality assessment and therefore the augmentation of safe MHA use on a broad scale. A challenge in this research is that the sequence in which apps are presented in the app store is incomprehensible and differs depending on which account is used for the search. In future studies, a web crawler could be used to search European app stores with keywords in order to build an unbiased database of available MHAs. Such a database already exists in China, and it contains all MHAs available in the United States, China, Japan, Brazil, and Russia [49].

Future studies should also shed light on the correlation between real-life user behavior and MARS or MARS-G ratings. As the MARS and MARS-G capture app quality, they could help predict the ability of users to download and use digital resources. Such research has already been conducted for ENLIGHT and real-life user engagement [50]. The efficacy of MHAs is strongly related to user adherence [50-52]; thus, high-quality apps might need to include adherence facilitation strategies to reach their potential.

Moreover, patient involvement should be taken into account. The user version of the MARS (uMARS) [53] should be translated and tested for reliability and validity as well, so that expert ratings of the MARS-G can be complemented with user ratings of the uMARS-G in German-speaking countries. In addition, there is a need for additional studies in the future to investigate the MARS-G and uMARS-G for apps related to specific health problems.

In conclusion, the MARS-G could be used by various stakeholders, such as public health authorities, patient organizations, researchers, health care providers (eg, physicians and psychotherapists), and interested third parties, to assess MHA quality. Furthermore, app developers could use the MARS-G as a tool to improve the quality of their apps.

## Appendix

Multimedia Appendix 1 Item correlation matrix of MARS and MARS-G.

Multimedia Appendix 2 Mobile Application Rating Scale-German.

## Abbreviations

ENLIGHT: Evaluation Tool for Mobile and Web-Based eHealth Interventions
ICC: intraclass correlation coefficient
LCRC: latent class reliability coefficient
MARS: Mobile App Rating Scale
MARS-G: German version of the Mobile App Rating Scale
MHA: mobile health app
MS: Molenaar-Sijtsma method
MSA: Mokken scale analysis
uMARS: user version of the Mobile App Rating Scale
