# Supplementary material for: The German Version of the Mobile App Rating Scale (MARS-G): Development and Validation Study
Source: JMIR Mhealth Uhealth. 2020 Mar 27;8(3):e14479. doi: 10.2196/14479 (PMC7148545; doi:10.2196/14479)
Supplement: Multimedia Appendix 1 [file mhealth_v8i3e14479_app1.docx]

| ***r*** | sa01  ger | sa02  ger | sa03  ger | sa04  ger | sa05  ger | sb01  ger | sb02  ger | sb03  ger | sb04  ger |
| --- | --- | --- | --- | --- | --- | --- | --- | --- | --- |
| sa01 | 0.96 | 0.79 | 0.43 | 0.44 | 0.51 | 0.27 | 0.19 | 0.26 | 0.37 |
| sa02 | 0.79 | 0.93 | 0.47 | 0.4 | 0.62 | 0.39 | 0.36 | 0.46 | 0.6 |
| sa03 | 0.47 | 0.49 | 0.96 | 0.68 | 0.48 | 0.34 | 0.19 | 0.34 | 0.32 |
| sa04 | 0.40 | 0.36 | 0.62 | 0.92 | 0.39 | 0.26 | 0.25 | 0.26 | 0.32 |
| sa05 | 0.54 | 0.66 | 0.48 | 0.49 | 0.86 | 0.39 | 0.39 | 0.51 | 0.54 |
| sb01 | 0.19 | 0.29 | 0.31 | 0.28 | 0.42 | 0.94 | 0.65 | 0.61 | 0.62 |
| sb02 | 0.14 | 0.29 | 0.18 | 0.23 | 0.46 | 0.62 | 0.96 | 0.78 | 0.80 |
| sb03 | 0.15 | 0.35 | 0.22 | 0.24 | 0.46 | 0.60 | 0.75 | 0.93 | 0.81 |
| sb04 | 0.29 | 0.52 | 0.34 | 0.38 | 0.56 | 0.67 | 0.78 | 0.82 | 0.95 |
| sc01 | 0.62 | 0.51 | 0.47 | 0.41 | 0.50 | 0.49 | 0.38 | 0.35 | 0.41 |
| sc02 | 0.62 | 0.55 | 0.48 | 0.35 | 0.53 | 0.44 | 0.22 | 0.26 | 0.35 |
| sc03 | 0.65 | 0.55 | 0.51 | 0.42 | 0.56 | 0.35 | 0.23 | 0.29 | 0.37 |
| sd01 | 0.35 | 0.38 | 0.40 | 0.37 | 0.54 | 0.10 | 0.08 | 0.14 | 0.23 |
| sd02 | 0.33 | 0.28 | 0.54 | 0.46 | 0.61 | 0.30 | 0.13 | 0.27 | 0.22 |
| sd03 | 0.42 | 0.48 | 0.19 | 0.09 | 0.49 | 0.29 | 0.14 | 0.28 | 0.35 |
| sd04 | 0.61 | 0.48 | 0.15 | -0.04 | 0.35 | 0.23 | 0.08 | 0.24 | 0.26 |
| sd05 | 0.66 | 0.52 | 0.72 | 0.16 | 0.71 | 0.44 | 0.30 | 0.59 | 0.47 |
| sd06 | -0.07 | 0.14 | 0.17 | 0.23 | 0.24 | 0.27 | 0.47 | 0.33 | 0.42 |
| sd07 | NA | NA | NA | NA | NA | NA | NA | NA | NA |

Note: Correlation estimates between MARS and MARS-G on item level part 1; NA: not applicable (due to the number of observations)

| ***r*** | sc01  ger | sc02  ger | sc03  ger | sd01  ger | sd02  ger | sd03  ger | sd04  ger | sd05  ger | sd06  ger |
| --- | --- | --- | --- | --- | --- | --- | --- | --- | --- |
| sa01 | 0.67 | 0.61 | 0.67 | 0.35 | 0.31 | 0.57 | 0.63 | 0.23 | -0.10 |
| sa02 | 0.6 | 0.54 | 0.57 | 0.38 | 0.42 | 0.59 | 0.49 | 0.35 | 0.13 |
| sa03 | 0.53 | 0.53 | 0.54 | 0.43 | 0.55 | 0.40 | 0.23 | 0.50 | 0.16 |
| sa04 | 0.37 | 0.31 | 0.41 | 0.30 | 0.40 | 0.22 | -0.06 | -0.08 | 0.28 |
| sa05 | 0.58 | 0.57 | 0.61 | 0.45 | 0.56 | 0.54 | 0.41 | 0.43 | 0.31 |
| sb01 | 0.51 | 0.43 | 0.39 | 0.12 | 0.23 | 0.31 | 0.26 | 0.55 | 0.28 |
| sb02 | 0.38 | 0.22 | 0.26 | 0.13 | 0.09 | 0.26 | 0.12 | 0.45 | 0.42 |
| sb03 | 0.35 | 0.20 | 0.25 | 0.15 | 0.20 | 0.33 | 0.23 | 0.27 | 0.26 |
| sb04 | 0.49 | 0.34 | 0.38 | 0.28 | 0.34 | 0.50 | 0.27 | 0.44 | 0.35 |
| sc01 | 0.90 | 0.84 | 0.85 | 0.35 | 0.32 | 0.51 | 0.37 | 0.59 | 0.13 |
| sc02 | 0.80 | 1.00 | 0.85 | 0.34 | 0.26 | 0.49 | 0.43 | 0.67 | 0.05 |
| sc03 | 0.79 | 0.86 | 0.97 | 0.38 | 0.34 | 0.50 | 0.42 | 0.70 | 0.10 |
| sd01 | 0.41 | 0.33 | 0.4 | 0.96 | 0.52 | 0.64 | 0.37 | -0.03 | 0.14 |
| sd02 | 0.42 | 0.32 | 0.41 | 0.57 | 0.88 | 0.45 | 0.34 | 0.43 | 0.34 |
| sd03 | 0.49 | 0.38 | 0.41 | 0.58 | 0.30 | 0.93 | 0.68 | 0.56 | 0.02 |
| sd04 | 0.48 | 0.44 | 0.48 | 0.35 | 0.25 | 0.61 | 0.92 | 0.45 | -0.04 |
| sd05 | 0.69 | 0.76 | 0.70 | 0.14 | 0.07 | 0.48 | 0.41 | 0.86 | 0.50 |
| sd06 | 0.09 | 0.05 | 0.14 | 0.15 | 0.37 | 0.17 | 0.02 | 0.61 | 0.99 |
| sd07 | NA | NA | NA | NA | NA | NA | NA | NA | NA |

Note: Correlation estimates between MARS and MARS-G on item level part 2; NA: not applicable (due to the number of observations)

| ***n*** | sa  01  ger | sa  02  ger | sa  03  ger | sa004  ger | sa005  ger | sb001  ger | sb  02  ger | sb  03  ger | sb  04  ger | sc  01  ger | sc  02  ger | sc  03  ger | sd  01  ger | sd  02  ger | sd  003  ger | sd  04  ger | sd  05  ger | sd  06  ger | sd  07  ger |
| --- | --- | --- | --- | --- | --- | --- | --- | --- | --- | --- | --- | --- | --- | --- | --- | --- | --- | --- | --- |
| sa  01 | 103 | 103 | 103 | 102 | 101 | 104 | 103 | 103 | 103 | 104 | 104 | 104 | 103 | 73 | 70 | 69 | 16 | 94 | 1 |
| sa  02 | 103 | 103 | 103 | 102 | 101 | 104 | 103 | 103 | 103 | 104 | 104 | 104 | 103 | 73 | 70 | 69 | 16 | 94 | 1 |
| sa  03 | 103 | 103 | 103 | 102 | 101 | 104 | 103 | 103 | 103 | 104 | 104 | 104 | 103 | 73 | 70 | 69 | 16 | 94 | 1 |
| sa  04 | 103 | 103 | 103 | 102 | 101 | 104 | 103 | 103 | 103 | 104 | 104 | 104 | 103 | 73 | 70 | 69 | 16 | 94 | 1 |
| sa  05 | 101 | 101 | 101 | 100 | 101 | 102 | 101 | 101 | 101 | 102 | 102 | 102 | 101 | 72 | 69 | 68 | 16 | 92 | 1 |
| sb  01 | 102 | 102 | 102 | 101 | 100 | 103 | 103 | 103 | 103 | 103 | 103 | 103 | 102 | 72 | 69 | 68 | 16 | 93 | 1 |
| sb  02 | 102 | 102 | 102 | 101 | 100 | 103 | 103 | 103 | 103 | 103 | 103 | 103 | 102 | 72 | 69 | 68 | 16 | 93 | 1 |
| sb  03 | 102 | 102 | 102 | 101 | 100 | 103 | 103 | 103 | 103 | 103 | 103 | 103 | 102 | 72 | 69 | 68 | 16 | 93 | 1 |
| sb  04 | 102 | 102 | 102 | 101 | 100 | 103 | 103 | 103 | 103 | 103 | 103 | 103 | 102 | 72 | 69 | 68 | 16 | 93 | 1 |
| sc  01 | 103 | 103 | 103 | 102 | 101 | 104 | 103 | 103 | 103 | 104 | 104 | 104 | 103 | 73 | 70 | 69 | 16 | 94 | 1 |
| sc  02 | 103 | 103 | 103 | 102 | 101 | 104 | 103 | 103 | 103 | 104 | 104 | 104 | 103 | 73 | 70 | 69 | 16 | 94 | 1 |
| sc  03 | 103 | 103 | 103 | 102 | 101 | 104 | 103 | 103 | 103 | 104 | 104 | 104 | 103 | 73 | 70 | 69 | 16 | 94 | 1 |
| sd  01 | 102 | 102 | 102 | 101 | 100 | 103 | 102 | 102 | 102 | 103 | 103 | 103 | 103 | 73 | 70 | 69 | 16 | 94 | 1 |
| sd  02 | 71 | 70 | 70 | 69 | 70 | 71 | 70 | 70 | 70 | 71 | 71 | 71 | 71 | 67 | 46 | 45 | 9 | 66 | 1 |
| sd  03 | 69 | 68 | 68 | 68 | 68 | 69 | 68 | 68 | 68 | 69 | 69 | 69 | 69 | 48 | 68 | 67 | 14 | 66 | 0 |
| sd  04 | 69 | 69 | 69 | 69 | 68 | 70 | 69 | 69 | 69 | 70 | 70 | 70 | 70 | 48 | 69 | 68 | 14 | 67 | 0 |
| sd  05 | 17 | 17 | 17 | 17 | 17 | 17 | 17 | 17 | 17 | 17 | 17 | 17 | 17 | 9 | 15 | 15 | 15 | 15 | 0 |
| sd  06 | 92 | 92 | 92 | 91 | 90 | 93 | 93 | 93 | 93 | 93 | 93 | 93 | 93 | 66 | 64 | 63 | 16 | 89 | 1 |
| sd  07 | 1 | 1 | 1 | 1 | 1 | 1 | 1 | 1 | 1 | 1 | 1 | 1 | 1 | 1 | 0 | 0 | 0 | 1 | 1 |

Note: number of included observations

| ***p*** | sa01  ger | sa02  ger | sa03  ger | sa04  ger | sa05  ger | sb01  ger | sb02  ger | sb03  ger | sb04  ger |
| --- | --- | --- | --- | --- | --- | --- | --- | --- | --- |
| sa01 | <.001 | <.001 | .002 | .001 | <.001 | .657 | >.999 | .947 | .026 |
| sa02 | <.001 | <.001 | <.001 | .007 | <.001 | .009 | .029 | <.001 | <.001 |
| sa03 | <.001 | <.001 | <.001 | <.001 | <.001 | .056 | >.999 | .067 | .139 |
| sa04 | .006 | .029 | <.001 | <.001 | .01 | .794 | >.999 | .859 | .155 |
| sa05 | <.001 | <.001 | <.001 | <.001 | <.001 | .008 | .011 | <.001 | <.001 |
| sb01 | >.999 | .357 | .243 | .509 | .003 | <.001 | <.001 | <.001 | <.001 |
| sb02 | >.999 | .343 | >.999 | >.999 | <.001 | <.001 | <.001 | <.001 | <.001 |
| sb03 | >.999 | .052 | >.999 | >.999 | <.001 | <.001 | <.001 | <.001 | <.001 |
| sb04 | .386 | <.001 | .063 | .014 | <.001 | <.001 | <.001 | <.001 | <.001 |
| sc01 | <.001 | <.001 | <.001 | .005 | <.001 | <.001 | .012 | .052 | .003 |
| sc02 | <.001 | <.001 | <.001 | .060 | <.001 | .001 | >.999 | .745 | .049 |
| sc03 | <.001 | <.001 | <.001 | .003 | <.001 | .042 | >.999 | .376 | .026 |
| sd01 | .044 | .018 | .006 | .025 | <.001 | >.999 | >.999 | >.999 | >.999 |
| sd02 | .515 | >.999 | <.001 | .011 | <.001 | >.999 | >.999 | >.999 | >.999 |
| sd03 | .054 | .006 | >.999 | >.999 | .004 | >.999 | >.999 | >.999 | .455 |
| sd04 | <.001 | .006 | >.999 | >.999 | .468 | >.999 | >.999 | >.999 | >.999 |
| sd05 | .468 | >.999 | .148 | >.999 | .196 | >.999 | >.999 | >.999 | >.999 |
| sd06 | >.999 | >.999 | >.999 | >.999 | >.999 | .833 | <.001 | .192 | .005 |
| sd07 | NA | NA | NA | NA | NA | NA | NA | NA | NA |

Note: p-values adjusted for multiple testing, part 1. NA: not applicable (due to the number of observations)

|  | sc01  ger | sc02  ger | sc03  ger | sd01  ger | sd02  ger | sd03  ger | sd04  ger | sd05  ger | sd06  ger | sd07  ger |
| --- | --- | --- | --- | --- | --- | --- | --- | --- | --- | --- |
| sa01 | <.001 | <.001 | <.001 | .043 | .807 | <.001 | <.001 | >.999 | >.999 | NA |
| sa02 | <.001 | <.001 | <.001 | .012 | .037 | <.001 | .005 | >.999 | >.999 | NA |
| sa03 | <.001 | <.001 | <.001 | .001 | <.001 | .099 | >.999 | >.999 | >.999 | NA |
| sa04 | .021 | .184 | .004 | .304 | .06 | >.999 | >.999 | >.999 | .657 | NA |
| sa05 | <.001 | <.001 | <.001 | <.001 | <.001 | <.001 | .075 | >.999 | .374 | NA |
| sb01 | <.001 | .001 | .011 | >.999 | >.999 | >.999 | >.999 | >.999 | .657 | NA |
| sb02 | .016 | >.999 | .859 | >.999 | >.999 | >.999 | >.999 | >.999 | .006 | NA |
| sb03 | .052 | >.999 | >.999 | >.999 | >.999 | .684 | >.999 | >.999 | >.999 | NA |
| sb04 | <.001 | .063 | .012 | .583 | .379 | .002 | >.999 | >.999 | .079 | NA |
| sc01 | <.001 | <.001 | <.001 | .048 | .618 | .001 | .259 | >.999 | >.999 | NA |
| sc02 | <.001 | <.001 | <.001 | .071 | >.999 | .004 | .042 | .493 | >.999 | NA |
| sc03 | <.001 | <.001 | <.001 | .013 | .379 | .002 | .049 | .306 | >.999 | NA |
| sd01 | .003 | .088 | .005 | <.001 | <.001 | <.001 | .229 | >.999 | >.999 | NA |
| sd02 | .052 | .684 | .062 | <.001 | <.001 | .235 | >.999 | >.999 | .551 | NA |
| sd03 | .004 | .170 | .069 | <.001 | >.999 | <.001 | <.001 | >.999 | >.999 | NA |
| sd04 | .005 | .023 | .006 | .367 | >.999 | <.001 | <.001 | >.999 | >.999 | NA |
| sd05 | .302 | .069 | .235 | >.999 | >.999 | >.999 | >.999 | .006 | >.999 | NA |
| sd06 | >.999 | >.999 | >.999 | >.999 | .321 | >.999 | >.999 | >.999 | <.001 | NA |
| sd07 | NA | NA | NA | NA | NA | NA | NA | NA | NA | NA |

Note: p-values adjusted for multiple testing, part 2. NA: not applicable (due to the number of observations)
