# Supplementary material for: The German Version of the Mobile App Rating Scale (MARS-G): Development and Validation Study
Source: JMIR Mhealth Uhealth. 2020 Mar 27;8(3):e14479. doi: 10.2196/14479 (PMC7148545; doi:10.2196/14479)
Supplement: Multimedia Appendix 2 [file mhealth_v8i3e14479_app2.pdf]

This is a Multimedia Appendix to a full manuscript published in the J Med Internet Res. For full copyright and citation information see http://dx.doi.org/10.2196/jmir.14479

Mobile Anwendungen Rating Skala (MARS-German)

Einordung der App

Diese Sektion dient dazu, beschreibende und technische Informationen über die App zu sammeln. Bitte lesen Sie dazu die Beschreibung der App auf iTunes, Google Play, um diese Informationen zu erhalten.

App Name:

Bewertung dieser Version:

Bewertung aller Versionen:

Entwickler:

Anzahl der Bewertungen dieser Version:

Anzahl der Bewertungen aller Versionen:

Version:

Letztes update:

Kosten für die Basisversion:

Kosten für die erweiterte Version:

Plattform:

- □ iOS
- □ Android
- □ Windows Mobile
- □ Andere:_________________

Kurze Beschreibung:

Angliederung

□ Unbekannt □ Kommerziell □ Regierung □ Gemeinnützige Organisation (NGO) □ Universität

Fokus: Ziel der App (Mehrfachauswahl)

1. Verbesserung von Befindlichkeit, Wohlbefinden
2. Reduktion depressiver Symptome
3. Reduktion von Angst
4. Reduktion von Stress
5. Verbesserung der Emotionsregulation
6. Unterstützung bei Verhaltensänderungen
7. Reduktion von Suchtverhalten
8. Unterstützung bei der Erreichung individueller Ziele
9. Unterhaltung
10. Verbesserung des Sozialverhaltens
11. Förderung körperlicher Gesundheit
12. Andere:______________________

Theoretischer Hintergrund bei Interventionen (Mehrfachauswahl)

1. Verhaltenstherapie
2. Kognitive Verhaltenstherapie
3. (Verhaltenstherapie der zweiten Welle)
4. Verhaltenstherapie der dritten Welle
5. (z. B. Mindfulness-based Psychotherapie, Akzeptanz und Commitment, Schematherapie, CBASP, DBT)
6. Systemische Therapie
7. Psychodynamische Psychotherapie
8. Humanistische Therapie
9. Integrative Therapie
10. (z. B. Interpersonelle Therapie)

Andere:_________________________

This is a Multimedia Appendix to a full manuscript published in the J Med Internet Res. For full copyright and citation information see http://dx.doi.org/10.2196/jmir.14479

Methoden (Mehrfachauswahl)

1. EMDR-Methode (zur Behandlung der Posttraumatischen Belastungsstörung),
2. Hypnotherapie (zur Raucherentwöhnung und zur Mitbehandlung bei somatischen Erkrankungen)
3. Datenerhebung, Messungen
4. Monitoring, Tracking
5. Feedback
6. Information, Edukation
7. Tipps, Ratschläge
8. Verfolgen eigener Ziele
9. Strategien, Fertigkeiten, Training (z.B.: Problemlösen, Stressmanagement, etc.)
10. Entspannungsübungen
11. Atemübungen
12. Körperübungen (z.B.: Yoga, Pilates, PMR)
13. Achtsamkeit/ Dankbarkeit
14. Akzeptanz
15. Ressourcenorientierung
16. Exposition
17. Gamification
18. Serious Games
19. Alternativmedizinische Interventionselemente (Homöopathie, Akupressur, etc.)
20. Erinnerungen, Reminder, Verstärker, etc.
21. Maßgeschneiderte Interventionen (tailored interventions), Echtzeit-Feedback
22. Schulmedizin
23. Andere:_________________________

Altersgruppe (Mehrfachauswahl)

1. Kleinkinder (unter 6)
2. Kinder (6-12)
3. Jugendliche (13-17)
4. Junge Erwachsene (18-25)
5. Erwachsene (26-64)
6. Ältere Erwachsene (ab 65)
7. UnspezifiziertXX

Technische Aspekte der App

1. Austausch mit anderen ist möglich (Facebook, Whatsapp, Telegram etc.)
2. Verfügt über eine App-Gemeinschaft
3. Benötigt Internetzugang
4. Andere:________________________

Kategorien im App-Store

1. Lifestyle
2. Medizin
3. Gesundheit & Fitness
4. Andere: __________________

Einbettung in therapeutische Angebote

1. Keine
2. Kommunikation mit Behandlern ist über die App oder ein Webinterface möglich
3. Zuteilung von Modulen durch den Behandler ist möglich
4. Teilen von Inhalten (z.B.: Fragebogenergebnisse, Nutzerstatistiken,

This is a Multimedia Appendix to a full manuscript published in the J Med Internet Res. For full copyright and citation information see http://dx.doi.org/10.2196/jmir.14479

Einsatzbereich

1. Prävention
2. Behandlung
3. Rehabilitation
4. Nachsorge
5. Andere

Behandlungsart

□ Stand-Alone

□ Blended Care (BC)

□ Nicht anwendbar

Unterstützung

□ Guided synchron

□ Guided asynchron

□ Technically guided

□ Unguided

Zertifizierung

1. Keine
2. Entspricht dem Medizinproduktegesetz
3. Andere: __________________

Bearbeitungsfortschritt, etc.) mit dem Behandler ist möglich

5. Andere: __________________

Sicherheit und Datenschutz

O Ja O Nein Erlaubt Passwortnutzung

O Ja O Nein Erfordert einen Login

O Ja O Nein Verfügt über eine Datenschutzerklärung

O Ja O Nein Erfordert das aktive Bestätigen einer Einverständniserklärung

O Ja O Nein Hinweise zur Finanzierung/ Interessenskonflikten

O Ja O Nein Kontakt/Ansprechperson/ Impressum

O Ja O Nein Sicherheit der Datenübertragung

O Ja O Nein Ort der Datenspeicherung

O Ja O Nein Werden Daten mit Dritten geteilt

O Ja O Nein Notfallfunktionen sind vorhanden

O Ja O Nein Sicherheitsstrategien bei Handyverlust

O Ja O Nein Andere:_______________

This is a Multimedia Appendix to a full manuscript published in the J Med Internet Res. For full copyright and citation information see http://dx.doi.org/10.2196/jmir.14479

App Qualitätsbewertung

Das MARS Rating erfasst die Qualität von Apps auf 4 Dimensionen. Alle Fragen werden von 1 „Mangelhaft“ bis 5 „Exzellent“ bewertet. Bitte markieren Sie die Nummer die sie am zutreffendsten empfinden. Bitte benutzen Sie die Beschreibungen welche in jeder Kategorie zur Verfügung gestellt werden.

SECTION A

Engagement - Spaß, Interesse, individuell Anpassbarkeit, Interaktivität (z.B. sendet Alarme, Nachrichten, Erinnerungen, Feedback, Austausch mit anderen ist möglich), Zielgruppenspezifität

I. Unterhaltung: Ist die App unterhaltsam und lustig zu benutzen? Werden Strategien zur Steigerung des Engagements durch Unterhaltung (z.B.: Gamification) geboten?

1. Langweilig, gar nicht unterhaltsam
2. Überwiegend langweilig
3. OK, unterhaltsam genug um sich für eine kurze Zeit mit der App zu beschäftigen (< 5 Minuten)
4. Mittelmäßig lustig und unterhaltsam, würde einen User für einige Zeit unterhalten (5-10 Minuten)
5. Sehr unterhaltsam und lustig, regt zu mehrmaligen Benutzen an

II. Interesse: Ist die App interessant zu benutzen? Werden Strategien angewandt um das Engagement durch eine interessante Präsentation der Inhalte zu erhöhen?

1. Überhaupt nicht interessant
2. Überwiegend nicht interessant
3. OK, weder interessant noch uninteressant; Nutzer würden sich damit eine kurze Zeit beschäftigen (< 5 Minuten)
4. Mittelmäßig interessant; Nutzer würden sich damit einige Zeit beschäftigen (insgesamt 5-10 Minuten)
5. Überaus interessant, so dass man zur wiederholten Nutzung der App angeregt wird

III. Individuelle Anpassbarkeit: Können alle nötigen Einstellungen den eigenen Wünschen entsprechend angepasst werden und werden diese Anpassungen abgespeichert (z.B. Klänge, Inhalt und Benachrichtigungen)?

1 Individuelle Anpassungen sind nicht möglich oder es ist nötig, diese bei jeder Nutzung neu den eigenen Wünschen anzupassen
2 Einige wenige individuelle Anpassungen sind möglich, aber durch die nur wenigen Möglichkeiten ist die Funktionalität der App beeinträchtigt
3 Grundlegende Einstellungen können individuell angepasst werden, so dass die Funktionalität der App gewährleistet ist
4 Vielfältige Optionen zur individuellen Anpassbarkeit stehen zur Verfügung
5 Eine maßgeschneiderte Anpassung an die Charakteristika und Wünsche der Nutzer ist

This is a Multimedia Appendix to a full manuscript published in the J Med Internet Res. For full copyright and citation information see http://dx.doi.org/10.2196/jmir.14479

möglich und alle individuellen Anpassungen werden für zukünftige Nutzungen übernommen

IV. **Interaktivität: Werden Nutzereingaben, Feedback und Aufforderungen (Erinnerungen, Optionen zum Austausch mit anderen, Benachrichtigungen, etc.) gegeben? Bemerkung: Diese Funktionen müssen individuell anpassbar und nicht überfordernd sein.**

1. Keine interaktiven Funktionen und/oder keine Eingaben der Nutzer sind möglich
2. Unzureichende Interaktivität, oder Feedback, oder Optionen für Eingaben der User, limitierte Funktionalität
3. Grundlegende interaktive Funktionen, angemessene Funktionalität
4. Vielfältige interaktive Funktionen, Feedback und Optionen für Eingaben der Nutzer sind vorhanden
5. Interaktive Funktionen, Feedback und Optionen für Eingaben der Nutzer sind auf einem so hohen Niveau, dass man das Gefühl hat, die App stellt sich auf die Person des Nutzers ein

V. **Zielgruppe: Ist der Inhalt der App (visuelle Aufbereitung, Sprache, Design) passend um die Zielgruppe anzusprechen?**

1. Sehr unpassend/unklar/verwirrend
2. Eher unpassend/unklar/verwirrend
3. OK, aber nicht Zielgruppen orientiert. Kann unpassend/unklar/verwirrend sein
4. Zielgruppenspezifisch, mit kleinen Mängeln
5. Sehr zielgruppenspezifisch, keine Probleme

A: Engagement Mittelwert=_________________

SECTION B

Funktionalität - Funktionen der App, Usability, Navigation, logischer Aufbau und motorische, gestische Handhabung der App

VI. **Leistung: Wie akkurat, schnell funktionieren die Komponenten (Knöpfe, Menüs) und Funktionen der App?**

1. Die App funktioniert nicht. Keine oder unzureichende Antwort (z.B. Abbrüche, Fehler, nicht funktionierende Funktionen, etc.)
2. Einige Funktionen laufen, jedoch langsam oder größere technische Probleme.
3. App funktioniert im Allgemeinen. Einige technische Probleme oder gelegentlich langsam
4. Die App funktioniert insgesamt gut, mit kleineren, vernachlässigbaren Problemen
5. Die App arbeitet fehlerfrei und schnell; keine technischen Fehler, oder – falls zutreffend – enthält einen Fortschrittsbalken

VII. **Usability: Wie leicht ist es den Umgang mit der App zu erlernen? Wie klar sind die Benennungen der Menüs, Symbole und Instruktionen?**

1. Keine oder unzureichende Erklärungen; Menübeschriftungen und Symbole sind verwirrend und/oder kompliziert
2. Nach hohem Aufwand benutzbar
3. Nach einigem Aufwand benutzbar
4. Benutzung ist leicht erlernbar oder die App weist klare Instruktionen zur Benutzung auf
5. Eine intuitive und einfache Benutzung der App ist sofort möglich

VIII. **Navigation: Ist die Menüführung logisch, akkurat, passend und wird nicht unterbrochen; sind alle notwendigen Verbindungen zwischen den Sektionen vorhanden?**

1. Verschiedene Abschnitte wirken unzusammenhängend/zufällig/verwirrend. Die Navigation ist kompliziert
2. Nach geraumer Zeit und hohem Aufwand benutzbar
3. Nach einiger Zeit und einigem Bemühen benutzbar
4. Leicht zu bedienen; es fehlen vernachlässigbare Verknüpfungen
5. Sehr logisch, einfach, klar und intuitive Menüführung. Bietet Verknüpfungen zum schnellen Wechsel zwischen verschiedenen Bereichen/Menüpunkten an

IX. **Motorisches, gestisches Design: Sind Interaktionen (tippen, wischen, drücken, scrollen, zoomen) einheitlich und intuitiv über alle Komponenten und Ansichten?**

1. Überhaupt nicht einheitlich; verwirrend
2. Häufig nicht einheitlich; verwirrend
3. OK mit einigen uneinheitlichen/verwirrenden Elementen

This is a Multimedia Appendix to a full manuscript published in the J Med Internet Res. For full copyright and citation information see http://dx.doi.org/10.2196/jmir.14479

4. Häufig einheitlich/intuitiv mit vernachlässigbaren Problemen
5. Auf perfekte Weise einheitlich und intuitiv verständlich

B: Funktionalität Mittelwert=_________________

This is a Multimedia Appendix to a full manuscript published in the J Med Internet Res. For full copyright and citation information see http://dx.doi.org/10.2196/jmir.14479

SECTION C

Ästhetik - Graphisches Design, visueller Anreiz, farbliche Gestaltung und stilistische Einheit

X. Layout: Sind das Arrangement und die Größe der Knöpfe/Symbole/Menüführung/ Inhalt passend oder entsprechend zoombar?

1. Mangelhaftes Design – unordentlich/ungeordnet; einige Optionen können nicht ausgewählt, gefunden, gesehen oder gelesen werden; Bildschirm wird nicht optimal genutzt
2. Schwaches Design - nicht einheitlich, unklar; einige Optionen sind schwierig auszuwählen, zu finden, zu sehen und zu lesen.
3. Mittelmäßige Qualität des Designs - einige Probleme Optionen auszuwählen, zu finden, zu sehen und zu lesen.
4. Gutes Design - überwiegend klar; Optionen können ausgewählt, gefunden, gesehen und gelesen werden.
5. Professionelles Design - einfach, klar, übersichtlich, logisch organisiert; Bildschirm wird optimal genutzt; jede einzelne Komponente des Designs erfüllt seinen Zweck

XI. Grafik: Wie hoch ist die Qualität und Auflösung der Grafiken von Knöpfen, Symbolen, Menüführung und Inhalten?

1. Grafiken wirken amateurhaft, schlechtes visuelles Design - völlig unproportional, stilistisch nicht einheitlich
2. Niedrige Qualität (z.B. Auflösung) der Grafiken. Niedrige Qualität des visuellen Designs - unproportional und stilistisch nicht einheitlich
3. Mittelmäßige Qualität der Grafiken und des visuellen Designs - überwiegend einheitlich im Design
4. Hohe Qualität der Grafiken und gute Auflösung des visuellen Designs - überwiegend proportional und einheitlich im Stil
5. Sehr hohe Qualität der Grafiken und sehr gute Auflösung des visuellen Designs - durchgehend proportional und einheitlich im Stil

XII. Visueller Anreiz: Wie gut sieht die App aus?

1. Kein visueller Anreiz - unangenehm zu betrachten, schwaches Design, beißende oder nicht zusammen passende Farben
2. Wenig visueller Anreiz - schlechtes Design, schlechter Farbgebrauch, visuell langweilig
3. Etwas visueller Anreiz - durchschnittlich weder angenehm noch unangenehm
4. Hoher visueller Anreiz - makellose Grafiken, einheitliches und professionelles Design
5. Wie in Punkt 4 beschrieben, darüber hinaus: Überaus großer visueller Anreiz - einprägsam, sticht positiv heraus; Farbgestaltung wirkt sich positiv auf die Optionen und die Menüführung der App aus

C: Ästhetik Mittelwert=__________________

This is a Multimedia Appendix to a full manuscript published in the J Med Internet Res. For full copyright and citation information see http://dx.doi.org/10.2196/jmir.14479

SECTION D

Information - Beinhaltet Informationen mit hoher Güte, Qualität (z. B. Texte, Feedback, Messinstrumente, Messergebnisse, Hinweise auf Literatur zum Vertiefen) aus einer glaubwürdigen Quelle. Bitte wählen Sie N/A (nicht anwendbar) aus, wenn der Punkt unzutreffend ist.

XIII. Genauigkeit der App-Beschreibung aus dem App-Store: Beinhaltet die App alle angegebenen Funktionen?

1. Irreführend - die App beinhaltet nicht die beschriebenen Komponenten/Funktionen oder hat keine Beschreibung
2. Überwiegend nicht korrekt - die App beinhaltet nur wenige der beschriebenen Komponenten/Funktionen
3. OK - die App beinhaltet einige der beschriebenen Komponenten, Funktionen.
4. Überwiegend korrekt - die App beinhaltet die meisten der beschriebenen Komponenten/Funktionen
5. Völlig korrekte Beschreibung der App sowie deren Komponenten/Funktionen

XIV. Ziele: Weist die App spezifische, messbare und erreichbare Ziele auf (spezifiziert in der Beschreibung im App Store oder in der App selbst)

N/A Die Beschreibung enthält keine Ziele, oder die Ziele der App sind nicht relevant für Ziele der Forschung

1 Die App kann die beschriebenen Ziele nicht erreichen

2 In der Beschreibung sind einige Ziele aufgelistet, aber die App kann diese nur mit einer geringen Wahrscheinlichkeit erreichen

3 OK. Die App hat klare Ziele, die erreicht werden könnten.

4 Die App hat klar spezifizierte Ziele, die messbar und erreichbar sind

5 Die App hat klar spezifizierte und messbare Ziele, die mit einer hohen Wahrscheinlichkeit erreicht werden können

XV. Qualität der Information: Ist der Inhalt korrekt, gut verfasst, relevant für das Ziel und das Thema der App?

N/A Die App enthält keine Informationen

1. Irrelevant, ungeeignet, unzusammenhängend, nicht korrekt
2. Kaum relevant, kaum geeignet, kaum zusammenhängend, möglicherweise nicht korrekt
3. Mittelmäßig relevant, mittelmäßig geeignet, mittelmäßig zusammenhängend, wirkt korrekt
4. Relevant, geeignet, zusammenhängend, korrekt
5. Sehr Relevant, sehr geeignet, sehr zusammenhängend, sehr korrekt

XVI. Quantität der Informationen: Ist der Inhalt der App umfassend und dennoch prägnant?

N/A: App beinhaltet keine Information

1. Minimal oder überfordernd
2. Unzureichend oder möglicherweise überfordernd
3. Ok, aber weder umfangreich noch prägnant
4. Bietet eine große Menge an Informationen, hat Lücken oder unnötige Details oder bietet keine weiteren Hinweise auf Literatur zum Vertiefen
5. Umfangreich und prägnant; beinhaltet Hinweise auf Literatur zum Vertiefen

XVII. Visuelle Informationen: Sind die visuellen Erklärungen von Konzepten – anhand von Tabellen, Graphiken, Bildern, Videos etc. – klar, logisch, korrekt?

N/A: App beinhaltet keine visuellen Information (beinhaltet z.B. nur Audio-Dateien oder Text)

1. Vollkommen unklar/verwirrend/falsch oder visuelle Informationen sind eigentlich nötig, fehlen aber
2. Überwiegend unklar/verwirrend/falsch
3. Ok, aber oft unklar/verwirrend/falsch
4. Meistens klar, logisch und richtig, mit unerheblichen/vernachlässigbaren Problemen
5. Perfekt klar, logisch und richtig

XVIII. Glaubwürdigkeit: Kommt die App aus einer legitimen Quelle (ist dies aus der Appstore-Beschreibung oder in der App selbst zu entnehmen)?

1. Die Quelle ist bekannt aber die Legitimität/Glaubwürdigkeit der Quelle ist fragwürdig (z.B. Werbe/Handelsbranche mit eigennützigem Interesse)
2. Scheint von einer legitimen Quelle zu kommen, kann aber nicht nachgeprüft werden (z.B. keine Webseite)
3. Entwickelt von einer kleinen gemeinnützigen Organisation (NGO: z. B. Krankenhaus, Zentrum etc.) oder von einer spezialisierten wirtschaftlichen Firma oder von Fördermittelgeber
4. Von der Regierung, einer Universität oder einer ähnlichen Institution entwickelt
5. Entwickelt mit Hilfe von wettbewerbsorientierten Regierungs- oder Forschungsgeldern (z.B. DFG, BMBF)

XIX. Evidenzbasierung: Wurde die App geprüft/getestet (muss nachweisbar sein z. B. durch wissenschaftliche Publikation)?

N/A: App wurde nicht geprüft/getestet

1. Die Evidenz zeigt auf, dass die App nicht funktioniert
2. Die App wurde getestet (z.B. Akzeptanz, Benutzerfreundlichkeit, Zufriedenheitsratings) und zeigt teilweise positive Ergebnisse in nicht randomisiert-kontrollierten Studien oder es gibt wenig bzw. keine widersprüchliche Evidenz.

3. Die App wurde getestet (z.B. Akzeptanz, Benutzerfreundlichkeit, Zufriedenheitsratings) und zeigt positive Ergebnisse in nicht-randomisierten Studien, es besteht keine widersprüchliche Evidenz
4. App wurde getestet und die Resultate aus 1-2 randomisiert-kontrollierten Studien weisen auf positive Ergebnisse hin
5. App wurde getestet und die Resultate aus ≥ 3 hochqualitativen randomisiert-kontrollierten Studien weisen auf positive Ergebnisse hin

D. Information Mittelwert=_________________*

* Fragen die mit „Nicht verfügbar / keine Antwort“ geratet wurden, nicht in die Mittelwert-Berechnung miteinbeziehen.

This is a Multimedia Appendix to a full manuscript published in the J Med Internet Res. For full copyright and citation information see http://dx.doi.org/10.2196/jmir.14479

SECTION PT

Psychotherapie – Diese Sektion erfasst Gütekriterien von Apps in Hinblick auf Patientensicherheit, Sinnhaftigkeit der Darbietung als App sowie die Güte der therapeutischen Angebote.

XX. Gewinn für Patienten

1. Die App kann den Patienten bei der Behandlung seiner Symptome/Erkrankung sehr schlecht unterstützen.
2. Die App kann den Patienten bei der Behandlung seiner Symptome/Erkrankung schlecht unterstützen.
3. Die App kann den Patienten bei der Behandlung seiner Symptome/Erkrankung teilweise unterstützen.
4. Die App kann den Patienten bei der Behandlung seiner Symptome/Erkrankung gut unterstützen.
5. Die App kann den Patienten bei der Behandlung seiner Symptome/Erkrankung sehr gut unterstützen.

XXI. Gewinn für Therapeuten

* N/A: Nur bei blended-care Ansätzen anwendbar

1. Die App bietet nichts, was der Therapeut zur Optimierung der Therapie nutzen kann.
2. Die App bietet wenig, was der Therapeut zur Optimierung der Therapie nutzen kann.
3. Die App bietet einiges, was der Therapeut zur Optimierung der Therapie nutzen kann.
4. Die App bietet viel, was der Therapeut zur Optimierung der Therapie nutzen kann.
5. Die App bietet sehr viel, was der Therapeut zur Optimierung der Therapie nutzen kann.

XXII. Mögliche Risiken, Nebenwirkungen und schädliche Effekte[1]

[1] Von der Benutzung von Apps die hier einen Wert unter 3 erreichen wird dringend abgeraten.

1. Die App ist aus therapeutischer Sicht schädlich. Es besteht ein hohes Risiko für unerwünschte Wirkungen (z.B.: irreführende Ratschläge, schädigende Übungen, unterlassene Hilfe, etc.).
2. Die App ist aus therapeutischer Sicht fragwürdig. Es besteht ein gewisses Risiko für unerwünschte Wirkungen (z.B.: falsche Rückmeldungen zu Fragebogenwerten, unkorrekte Informationen und Empfehlungen, etc.).
3. Die App ist aus therapeutischer Sicht teilweise sicher. In Hinblick auf best practice Richtlinien zur Behandlung der jeweiligen Erkrankung bestehen Mängel (z.B.: keine Notfallfunktion, etc.).
4. Die App ist aus therapeutischer Sicht vorwiegend sicher. In Hinblick auf best practice Richtlinien zur Behandlung der jeweiligen Erkrankung bestehen nur minimale Mängel.

This is a Multimedia Appendix to a full manuscript published in the J Med Internet Res. For full copyright and citation information see http://dx.doi.org/10.2196/jmir.14479

5. Die App ist aus therapeutischer Sicht sicher und entspricht den aktuellen best practice Richtlinien der jeweiligen Erkrankung.

XXIII. Übertragbarkeit in die Routineversorgung

1. Die Evidenz zeigt auf, dass die App nicht funktioniert bzw. es ist keine Information auffindbar.
2. die App wurde nicht an Patienten getestet (z. B. Probanden mit erhöhten Werten psychischer Symptomatik in Fragebögen wurden untersucht ohne dass psychische Störungen diagnostiziert wurden) sowie nur unter Bedingungen, die nicht repräsentativ für die psychotherapeutische Routine sind (z. B. von Studenten in universitären Settings)
3. die App wurde an Patienten getestet (z. B. Diagnose mittels SKID ermittelt oder Personen in psychotherapeutischer Behandlung), aber nur unter Bedingungen, die nicht repräsentativ für die psychotherapeutische Routine sind (z. B. nur in universitären Settings getestet)
4. die App wurde an Patienten getestet (z. B. Diagnose mittels SKID ermittelt oder Personen in psychotherapeutischer Behandlung) und die Bedingungen waren etwas repräsentativ für die psychotherapeutische Routine (z. B. nur in einer Klinik, nur in einer Psychotherapieambulanz)
5. die App wurde an Patienten getestet (z. B. Diagnose mittels SKID ermittelt oder Personen in psychotherapeutischer Behandlung) und die Bedingungen waren sehr repräsentativ für die psychotherapeutische Routine (z. B. in mehreren Kliniken, mehreren Psychotherapieambulanzen)

PT. Psychotherapie Mittelwert=_________________*

*Fragen die mit „Nicht verfügbar / keine Antwort“ geratet wurden, nicht in die Mittelwert-Berechnung miteinbeziehen.

This is a Multimedia Appendix to a full manuscript published in the J Med Internet Res. For full copyright and citation information see http://dx.doi.org/10.2196/jmir.14479

SECTION E (Subjektive Qualität)

XXIV. Würden Sie die App Personen empfehlen die davon profitieren könnten?

1. Überhaupt nicht: Ich würde die App niemanden empfehlen
2. Ich würde die App nur wenigen Personen empfehlen
3. Vielleicht: Ich würde die App schon einigen Personen empfehlen
4. Ich würde die App vielen Personen empfehlen
5. Auf jeden Fall: Ich würde die App jedem empfehlen

XXV. Wie viele Male würden Sie die App in den nächsten zwölf Monaten nutzen, falls diese relevant für Sie wäre?

1. Nie
2. 1-2
3. 3-10
4. 10-50
5. >50

XXVI. Würden Sie diese App erwerben, wenn sie kostenpflichtig wäre?

1. Nein
2. Vielleicht
3. Ja

XXVII. Wie viele Sterne würden Sie für die App insgesamt vergeben?

1. ★ Eine der schlechtesten Apps, die ich je genutzt habe
2. ★★
3. ★★★ Durchschnittlich
4. ★★★★
5. ★★★★★ Eine der besten Apps, die ich je genutzt habe

Bewertung

Bewertung der App-Qualität für Sektion

A. Engagement Mittelwert = ___________________

B. Funktionalität Mittelwert = ___________________

C. Ästhetik Mittelwert = ___________________

D. Information Mittelwert = ___________________

PT: Psychotherapie Mittelwert= ___________________

Mittelwert der App-Qualität = ___________________

Subjektive App- Qualität = ___________________

This is a Multimedia Appendix to a full manuscript published in the J Med Internet Res. For full copyright and citation information see http://dx.doi.org/10.2196/jmir.14479

SECTION F

Diese zusätzlichen Angaben erfassen die wahrgenommen Auswirkungen der App-Nutzung auf das Wissen, die Einstellung und Veränderungsintention sowie die Wahrscheinlichkeit einer tatsächlichen Veränderung des Verhaltens hin zum gewünschten Gesundheitsverhalten.

1. Bewusstsein: Die App steigert das Bewusstsein für die Wichtigkeit, sich mit dem Gesundheitsverhalten zu beschäftigen

Stimme überhaupt nicht zu Stimme sehr zu

1 2 3 4 5

2. Wissen: Die App erhöht das Verständnis und Wissen über das gewünschte Gesundheitsverhalten

Stimme überhaupt nicht zu Stimme sehr zu

1 2 3 4 5

3. Einstellung: Die App ändert die Einstellung, das Gesundheitsverhalten zu verbessern

Stimme überhaupt nicht zu Stimme sehr zu

1 2 3 4 5

4. Absicht zur Verhaltensänderung: Die App fördert die Absicht und Motivation, das Gesundheitsverhalten zu verbessern.

Stimme überhaupt nicht zu Stimme sehr zu

1 2 3 4 5

5. Suche nach Hilfe: Die App ermutigt dazu, sich weitere Hilfen bezüglich des Gesundheitsverhaltens (wenn erforderlich) zu suchen

Stimme überhaupt nicht zu Stimme sehr zu

1 2 3 4 5

6. Verhaltensänderung: Die App ermöglicht die gewünschte Änderung des Gesundheitsverhaltens

Stimme überhaupt nicht zu Stimme sehr zu

1 2 3 4 5
